# Effect of the Mediterranean diet and probiotic supplementation in the management of mild cognitive impairment: Rationale, methods, and baseline characteristics

**DOI:** 10.3389/fnut.2022.1037842

**Published:** 2022-12-08

**Authors:** Magdalena P. Cardelo, Andreea Corina, Ana Leon-Acuña, Gracia M. Quintana-Navarro, Juan F. Alcala-Diaz, Oriol Alberto Rangel-Zuñiga, Antonio Camargo, Cristina Conde-Gavilan, Claudia Carmona-Medialdea, Juan A. Vallejo-Casas, Elvira Carmona-Asenjo, Juan J. Ochoa-Sepulveda, Eduardo Aguera-Morales, Javier Delgado-Lista, Niki Katsiki, José Lopez-Miranda, Francisco Perez-Jimenez, Elena M. Yubero-Serrano, Pablo Perez-Martínez

**Affiliations:** ^1^Unidad de Gestión Clinica Medicina Interna, Lipids and Atherosclerosis Unit, Maimonides Institute for Biomedical Research in Córdoba (IMIBIC), Reina Sofía University Hospital, University of Córdoba, Córdoba, Spain; ^2^CIBER Physiopathology of Obesity and Nutrition (CIBEROBN), Institute of Health Carlos III, Madrid, Spain; ^3^Neurology Service, Maimonides Institute for Biomedical Research in Córdoba (IMIBIC), Reina Sofía University Hospital, Córdoba, Spain; ^4^Unidad de Gestión Clinica de Medicina Nuclear, Maimonides Institute for Biomedical Research in Córdoba (IMIBIC), Reina Sofía University Hospital, Córdoba, Spain; ^5^Department of Nutritional Sciences and Dietetics, International Hellenic University, Thessaloniki, Greece; ^6^School of Medicine, European University Cyprus, Nicosia, Cyprus

**Keywords:** mild cognitive impairment, Mediterranean diet, dietary strategies, probiotics, gut-brain axis

## Abstract

**Introduction:**

Mild cognitive impairment (MCI) can progress to Alzheimer’s disease (AD). When MCI is not properly controlled, the speed of deterioration can dramatically increase. Reduction of oxidative stress/inflammation and the modulation of the *gut-brain axis* could be new potential therapeutic targets for the prevention and treatment of AD. Consumption of specific nutrients, diets and probiotic supplementation have been evaluated for neurodegenerative disorders. We focus on a detailed description of the study methods and baseline characteristics of a clinical trial aiming to evaluate the efficacy of a combined nutritional intervention, i.e., a Mediterranean diet with probiotics, on cognitive capacity in a population with MCI.

**Methods:**

In this randomized, latin-square crossover, double-blind, and controlled dietary intervention trial (clinicaltrials.gov NCT05029765), 47 MCI patients were randomized to consume three dietary interventions for 24-weeks each: (1) A Mediterranean diet supplemented with probiotics (10^9^ colony-forming units of *Lactobacillus rhamnosus* and *Bifidobacterium longum*); (2) A Mediterranean diet + placebo; and (3) A Healthy diet according to the World Health Organization (WHO) recommendations. Participants will be evaluated before and after each of the three intervention periods (each 24-weeks, with a total of 72-weeks) for adherence to the assigned diet, blood tests, cognitive performance, gut microbiota analysis and functional neuroimaging studies.

**Results:**

Fifty patients, ≥60 years-old and diagnosed with MCI, underwent randomization. A total of 47 patients completed follow-up dietary interventions (57.4% males), with a good glycemic control (HbA1c 5.8 ± 0.1%, fasting glucose and insulin 99.7 ± 3.3 mg/dL and 10.4 ± 0.9 mU/L, respectively), elevated systolic blood pressure (136.9 ± 2.1 mmHg) and increased degree of inflammation (high-sensitivity C-reactive protein, 8.8 ± 0.9 mg/dL). Baseline adherence to the Mediterranean diet was medium (7.5 ± 0.3 points on the score that ranged from 0 to 14 points).

**Conclusion:**

The results of this clinical study would provide more evidence on the need for dietary therapeutic strategies, for clinical and individual practice, in the management of MCI patients to reduce the risk of AD development. Targeting lifestyle modifications in high-risk populations could prevent substantial cases of cognitive decline.

**Clinical trial registration:**

[ClinicalTrials.gov], identifier [NCT05029765].

## Introduction

Mild cognitive impairment (MCI) is usually defined as a transitional state between normal cognition and dementia (1). This prodromic state, which can often go undiagnosed, is characterized by a decline in cognitive function with a relatively intact daily living and social performance (2, 3). When this symptomatology is not properly controlled, the speed of deterioration can dramatically increase, progressing to dementia. The annual MCI progression rate to Alzheimer’s disease (AD), the most common cause of dementia (4), varies from 8.1% in clinical and 6.8% in community settings (5), suggesting that a large proportion of MCI patients do not progress to AD and may revert to normal cognition. Therefore, there is a need to establish preventive and effective strategies that may modulate MCI progression and reduce AD incidence.

The pathophysiological processes of the AD begin a decade or more before the clinical signs of the disease are detectable (6, 7). Accumulation of extracellular β-amyloid A plaques, intra-neuronal neurofibrillary tau tangles, neuronal and synaptic loss, neuro-inflammation and oxidative stress are the major neuropathological hallmarks of this disease (8–10). Moreover, recent evidence supports an interconnection between the gastrointestinal tract and the brain (the *gut-brain axis*), suggesting that alteration in the composition of the gut microbiota may also contribute to AD development, thus representing a potential therapeutic target for the prevention and treatment of AD (11, 12).

The failure of different clinical trials with candidate drugs to treat AD has refocused attention on the potential of lifestyle interventions in pre-symptomatic but high-risk individuals, such as in the case of MCI patients, to delay or prevent AD progression (13–15). Although several studies evaluated single nutrients and foods (16–18), the study of overall dietary patterns may provide a more powerful tool for assessing dietary habits, as well as the synergistic and cumulative effects of specific nutrients against these diseases. Results from recent clinical studies suggest that the adherence to a Mediterranean diet, characterized by high consumption of vegetables, fruits, legumes, nuts, wholes grains, olive oil (virgin or extra-virgin olive oil –VOO and EVOO, respectively) as the main fat (monounsaturated –MUFA fat) source, could be related to a reduced risk of developing chronic diseases as cognitive impairment and dementia (19–24). On the other hand, efficacy of probiotics administration, such as *Bifidobacterium* and *Lactobacillus* strains, have been evaluated for neurodegenerative disorders (25–27).

Considering all the above, the main aim of this trial is to evaluate the efficacy of a combined nutritional intervention, i.e., a Mediterranean diet rich in EVOO, supplemented with probiotics (10^9^ colony-forming units of *Lactobacillus rhamnosus* and *Bifidobacterium longum*), on cognitive capacity, measured by Alzheimer’s disease Assessment Scale-Cognitive (ADAs-Cog-11) (28) in a population with MCI, as a therapeutic strategy to prevent AD progression, based on the paradigm that changes in the gut microbiota induce biological mechanisms on the gut-brain-axis. The effect of this combined nutritional intervention will be compared with both the same Mediterranean diet without supplemented probiotics and a World Health Organization (WHO) diet (as a control diet).

In this report, we focus on a detailed description and analysis of the study methodology, including the dietary intervention, study participant selection, recruitment, and adherence strategies, so that these may be applied to future trials.

## Methods and design

### Overall design

This clinical study is a randomized, latin-square crossover, double-blind, and controlled dietary intervention trial performed in MCI patients, with an intention-to-treat analysis. The study was conducted at the Maimónides Biomedical Research Institute of Cordoba (IMIBIC, for its initials in Spanish) and the Reina Sofía University Hospital, where the screening, selection and recruitment of the patients who participated in the study were carried out. The study was registered at ClinicalTrials.gov (number NCT05029765). The study protocol was approved by the Human Investigation Review Committee of the Reina Sofía University Hospital, according to institutional and Good Clinical Practice guidelines.

The sample size was calculated based on the following assumptions: for the main outcome variable of the study (i.e., an improvement on the ADAS-Cog-11), a change of 20% from the baseline test was considered as significant (29); alpha risk: 0.05; difference in percentage between comparisons of 20%; power (1-ß):0.90; estimated losses: 10%; two-tailed contrast. Based on these premises, 41 patients were needed. With the aim of minimizing possible losses and increasing the study’s power, a total of 50 participants were included.

### Study population

The inclusion and exclusion criteria of diagnosed MCI patients are detailed in Table 1. To sum up, patients were eligible if they were ≥60 years-old and had:

**TABLE 1:** Inclusion and exclusion criteria of study patients.

Inclusion criteria
1. Patients with age ≥60 years old. 2. Informed consent: All participants were agree to being included in the study by signing the protocol approved by the Reina Sofía University Hospital Clinical Research Ethics Committee. In this written statement of consent, it was state that patients were be chosen for inclusion in the groups on a random basis. 3. Diagnostic criteria: Patients were diagnosed with MCI if they met the following criteria: (a) Clinical dementia rating (CRD) = 0.5 (30–32). (b) Mini mental examination de Folstein (MMSE) >23 (33). (c) Repeatable battery for the assessment of neuropsychological status (RBANS) -delayed memory subtest ≤85) (34, 35).
4. Geriatric depression scale (GDS) <6 (36). 5. Adequate visual and auditory abilities to perform neuropsychological testing. 6. Have an educational background during a minimum of 6 years or similar work history. 7. Have a family member or caregiver who could accompany the participant to clinical visits.

**Exclusion criteria**

1. Pharmacological treatment with an unstable dose and intake of probiotics within the 4 weeks before to screening (including psychotropic and other drugs affecting the alertness and cognitive capacity of the patients). 2. Any uncontrolled medical or neurological condition that could contribute to the cognitive capacity (e.g., substances abuse, vitamin B12 deficiency, abnormal thyroid function, stroke or other cerebral vascular disease, dementia with Lewy bodies or traumatic brain injury). 3. A clinically significant psychiatric illness (e.g., major depression, schizophrenia, or bipolar affective disorder) within the 6 months before to screening. 4. Transient ischemic attack or cerebrovascular accident or any unexplained loss of consciousness, within 1 year before to screening (in case of vascular deficit with cognitive sequelae that may still be reversible). 5. Poorly controlled diabetes mellitus [with values of glycosylated hemoglobin (HbA1c) >8%]. 6. History of unstable angina, myocardial infarction, chronic heart failure (Class 3 or 4 according New York Heart Association), within 1 year before to screening. 7. Uncontrolled hypertension defined as the mean of 3 measures of systolic blood pressure/diastolic blood pressure >165/100 mmHg, and persistent systolic blood pressure/diastolic blood pressure >180/100 mmHg, within the 3 months prior to randomization. 8. History of recurrent seizures within 10 years before the screening.
9. Use of alcohol of substance abuse, within 1 year before the screening.
10. Patients with other chronic diseases (e.g., severe psychiatric disease, chronic kidney failure, chronic liver disease, neoplastic disease, chronic obstructive pulmonary disease, endocrinopathies and digestive tract diseases.

• Clinical Dementia Rating (CDR) scale score = 0.5 (30–32).
• Mini Mental Examination de Folstein (MMSE) >23 (33).
• Repeatable Battery for the Assessment of Neuropsychological Status (RBANS)-delayed memory subtest ≤85 (34, 35).
• Geriatric Depression Scale score of <6 (confirming lack of mild or major depression) (36).
• Adequate visual and auditory abilities to carry out neuropsychological tests, a minimum educational background and a family member or caregiver who could accompany the participant to clinical visits.

**FIGURE 1:**
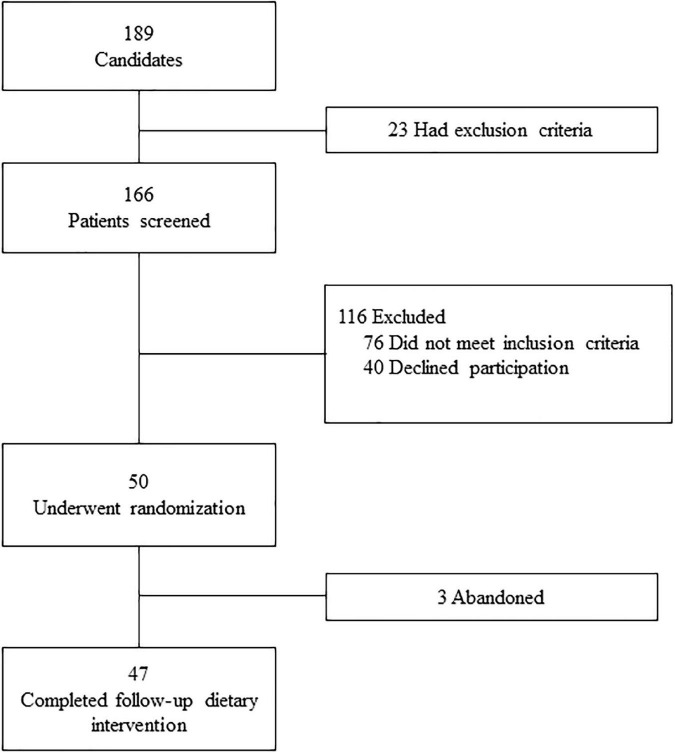
Screening, selection and recruitment flow-chart of patients for the study.

The screening, selection and recruitment processes were carried out by internists and neuropsychologists between January 2017 and September 2018 (Figure 1). Initially, out of 189 potentially eligible candidates, 166 were screened. From these patients, 116 were excluded (76 did not meet inclusion criteria and 40 declined participation). Finally, 50 patients, ≥60 years-old and diagnosed with MCI, underwent randomization. A total of 47 patients completed follow-up dietary intervention (three abandoned the dietary intervention and denied their permission to be followed up by electronic health record or phone calls, and therefore were censored at that point). All the patients gave their written informed consent to participate in the study.

### Dietary guidelines

**FIGURE 2:**
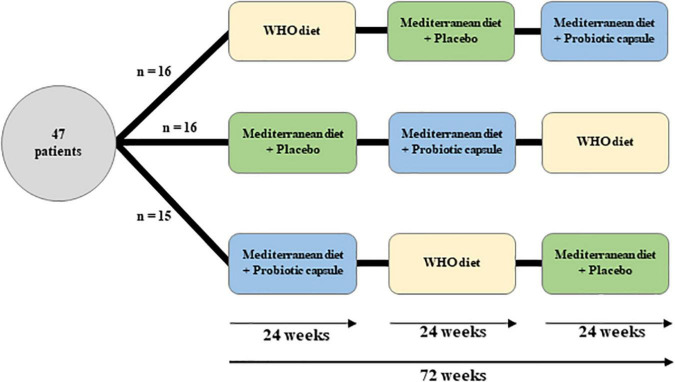
Study design.

Participants enrolled in the study were randomly assigned, by a computerized random sequence generator, to consume three dietary interventions, for 24-weeks each, with a dietary follow-up period of 72-weeks as a total (Figure 2): (1) A Mediterranean diet supplemented with probiotics [10^9^ colony-forming units of *Lactobacillus rhamnosus* CECT8361 and *Bifidobacterium longum* CECT737 -Biopolis-MIX42 (ADM Biopolis, Paterna, Valencia, Spain)]; (2) A Mediterranean diet + placebo; and (3) A Healthy diet according to WHO recommendations.

Safety studies as well as preclinical and/or clinical trials for each of the strains have been carried out following WHO criteria (37–39).

#### Dietary interventions

Dietary interventions were performed by a team of registered dietitians (RDs) who were previously trained to ensure uniformity and the quality of the intervention. The primary goal was to change the eating habits of the patients toward the randomized healthy diet (Mediterranean or WHO diet), focusing on the overall quality of the diet, rather than on specific nutrients, and to evaluate the additive effect of Mediterranean diet supplemented with probiotics. No intervention to increase physical activity or lose weight was included. Since the study patients were MCI-diagnosed, dietary recommendations were particularly focused on the family member or person responsible for cooking at home.

**FIGURE 3:**
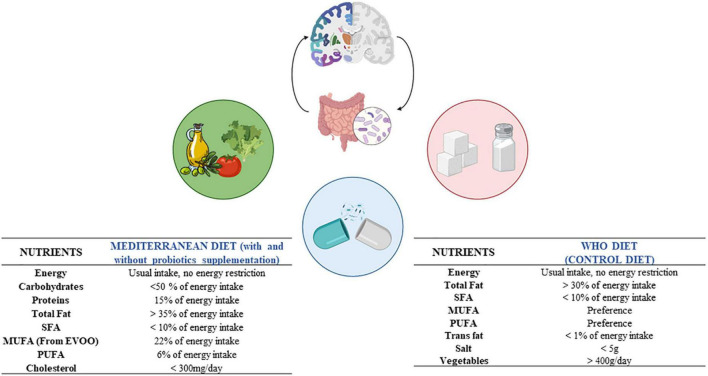
Nutrient composition of the dietary interventions analyzed in the study. WHO, world health organization; SFA, saturated fatty acids; MUFA, monounsaturated fatty acids; PUFA, polyunsaturated fatty acids.

The three dietary interventions included foods from all major food groups, but not total calorie restriction was advised. The Mediterranean diet (supplemented or not with probiotics) comprised a minimum of 35% of total calories from fat [22% MUFAs, 6% polyunsaturated fatty acids (PUFAs), <10% saturated fatty acids (SFAs)], ≤50% from carbohydrates and 15% from protein. The WHO diet included a minimum of 30% of total calories from fat (mainly from MUFAs and PUFAs), emphasizing on the reduction of SFAs (<10%), *trans*-fat (<1%) and salt consumption (<5 g), and an increase in vegetables and fruits (400 g/day) (Figure 3).

In the three dietary groups, RDs gave personalized counseling to achieve the goals for each diet progressively as follows:

– In both Mediterranean diet groups (with and without probiotics supplementation), patients were recommended to consume, as we previously described (40): (1) abundant use of EVOO for cooking and dressing (≥4 tablespoons/day; 10–15 g/tablespoon), (2) daily consumption of at least two servings of vegetables (200 g/serving; at least one serving raw or as salad) and three or more units of fresh fruit (125–150 g/unit), (3) weekly consumption of at least three servings of legumes (150 g cooked weight/serving), three or more servings of fish or seafood (especially oily fish; 100–150 g/serving) and fresh nuts and seeds (three or more handfuls per week), (4) cooking dishes seasoned with “sofrito” (a slow-cooked homemade sauce with tomato, garlic, onion, aromatic herbs, and olive oil) at least twice a week, (5) a reduction in meat consumption, choosing (skinless) white meat instead of red meat or processed meat (<1 serving/day), (6) avoidance of additional fats (butter, margarine, seed oils, creams, etc.) and foods rich in sugar and unhealthy fats (commercial bakery products, chips, precooked food, sugared beverages, etc.), and (7) in alcohol drinkers, a moderate consumption of red wine.
– In the WHO diet group, patients were recommended to: (1) consume unsaturated fats, as primary source of fat (found in fish, avocado, nuts, and vegetables oils) instead of saturated fats (found in fatty meat, butter, palm and coconut oil, cream, cheese) and industrially produced *trans*-fats (41, 42), (2) consume vegetable products as fruits, vegetables, legumes, nuts and whole grains (e.g., unprocessed maize, millet, oats, wheat, and brown rice) (42), (3) consume five portions (400 g) of fruit and vegetables daily, excluding potatoes, sweet potatoes, and other starchy roots, (4) reduce the intake of free sugars (<10% of total energy intake) found in foods or drinks by the manufacturer, cook or consumer, as well as sugars naturally present in honey, syrups, fruit juices and fruit juice concentrates (43), and (5) consume <5 g of salt (equivalent to about one teaspoon) per day (44). Salt should be iodized.

The RDs conducted each dietary intervention with the same intensity. Table 2 summarizes the frequency and type of visits performed during the intervention period. At baseline and every 12-weeks, patients had an individual face-to-face visit with the RDs which included assessment of dietary intake and adherence, feedback, and reinforcement, as well as future directions. At each visit, RDs and patients worked together to identify dietary habits that needed to be changed, to set short-term goals and to work out how to implement modifications. Between each face-to-face visit, telephone interviews were performed by the RDs to monitor compliance with the assigned diet, negotiate nutrition goals, and reinforce the dietary recommendations.

**TABLE 2:** Interventions, subsequent care and follow up visits.

Item/measurements	Brief description	BASELINE	Weeks											
			
			6	12	18	24	30	36	42	48	54	60	66	72
Eligibility questionnaire	Inclusion/exclusion criteria	1												
General questionnaire	Personal and family history, medical conditions, medications, blood pressure, smoking, alcohol intake, weight, height, waist circumference and other clinical scales	1												
Socio-economic questionnaire	Socio-demographic and economic characteristics, marital status, job, level of education	1												
Informed consent	Informed consent for the study and the Biobank store samples	1												
Randomization		1												
Follow-up questionnaire	Symptoms and conditions, marital status, job, medications, blood pressure, weight, waist circumference and other clinical scales			1		1		1		1		1		1
Tolerance questionnaire	Adverse experiences			1		1		1		1		1		1
Capsules administration		1		1		1		1		1		1		
**Neuropsychological status**														
Functional neuroimaging studies	^18^F-FDG-Positron emission tomography (PET)	1				1				1				1
Neuropsychological evaluation		1				1				1				1
ADAS-COG-11	Alzheimer’s disease Assessment scale–cognitive subscale (11-task version)	1				1				1				1
Neuropsychological test battery	More sensitive neuropsychological tests for specific domains (memory, attention, executive and visuospatial) and subdomains of cognition.	1				1				1				1
IDDD	Interview for deterioration of daily living in dementia (IDDD)	1				1				1				1
FAQ5	Physical functional ability questionnaire (FAQ5)	1				1				1				1
NPI-Q	Neuropsychiatric inventory–questionnaire (NPI-Q)	1				1				1				1
**Nutrition registers/questionaries’**														
3 Days register food consumption		1				1				1				1
Food frequency questionnaire (FFQ)	Validated 137-item FFQ	1				1				1				1
Dietary adherence assessment	Mediterranean diet adherence screener (MEDAS)	1		1		1		1		1		1		1
Dietary reinforcement	Face-to-face and telephone interviews	1	1	1	1	1	1	1	1	1	1	1	1	
**Samples collection**														
Blood sample	Lipids, glucose, renal function, blood count, and others	1				1				1				1
Urine sample		1				1				1				1
Stool sample		1				1				1				1

Written materials were designed and given to the patients at face-to-face sessions (every 12 weeks) to enhance oral recommendations: leaflets summarizing the main food components, their frequency of consumption, and cooking recipes focused on increasing skills for preparing meals complied with the assigned diet and meal plans. Telephone interviews between each face-to-face session were performed to enhance dietary adherence and solve possible doubts about given recommendations. To encourage dietary adherence, patients also received free EVOO during the 24-weeks period of both interventions with Mediterranean diets (with and without supplemented with probiotics) (approximately 1 L per week).

#### Dietary intake assessment

Information on habitual dietary intake was collected at baseline and at the end of each dietary intervention (i.e., 24, 48, and 72-weeks) using a 137-item semi-quantitative food-frequency questionnaire (FFQ), previously validated in the Spanish population (45, 46). Participants were asked to report their average intake of different food and beverage items over the previous 12 months, as we previously described (40). For each item, typical portion size was included, and consumption frequencies were registered in nine categories ranging from “never or hardly ever” to “≥six times/day.” Energy and nutrient intakes were calculated from Spanish food composition tables (47, 48).

A prospective 3 day-food record of 3 consecutive days [covering 2 weekdays and 1 weekend day (49, 50)] was also assessed at baseline and at the end of each dietary intervention (i.e., 24, 48, and 72-weeks) to enrich the information about dietary consumption and cooking methods.

#### Dietary adherence assessment

The 14-item MEditerranean Diet Adherence Screener (MEDAS) was used to measure adherence to the Mediterranean diet (40) in each dietary intervention (in both the Mediterranean dietary groups-with and without probiotics supplementation- and in the WHO dietary group-control diet). This score is an extension of a 9-point score developed by Martinez-Gonzalez et al. (51) and consists of two questions about eating habits, eight questions about the frequency of consumption of typical foods of the Mediterranean diet, and four questions on the consumption of foods not recommended in this diet. Each question was scored with 0 (non-compliant) or 1 (compliant), and the total score (from a total of 14 questions), can range from 0 to 14. Therefore, a score of 14 points depicts maximum adherence.

### Outcome measures

Primary and secondary outcomes of the study are summarized in Table 3. As part of the main objective, the following outcomes were assessed upon inclusion in the study (baseline), and at the end of each dietary intervention period (i.e., 24, 48, and 72-weeks) (Table 2).

**TABLE 3:** Description of the main and secondary objectives of the study.

Primary objective
1. Evaluate the efficacy of a combined nutritional intervention (a Mediterranean diet rich in EVOO, supplemented with probiotics (10^9^ colony-forming units of Lactobacillus rhamnosus and Bifidobacterium longum), on cognitive capacity, measured by Alzheimer’s disease Assessment Scale-Cognitive (ADAs-Cog-11).

**Secondary objectives**

1. Determine changes associated with the dietary intervention in the percentage of different families of gut microbiota to identify specific patterns and its effect on cognitive capacity.
2. Evaluate neuro-functional changes associated to the dietary intervention evaluated by 2-Deoxy-2-[fluorine-18] fluoro-D-glucose (^18^F-FDG) positron emission tomography (PET).
3. Evaluate if the dietary intervention produces changes in endotoxemia [lipopolysaccharide (LPS) and LPS-binding protein (LBP) levels] and its relationship with cognitive capacity.
4. Determine if the dietary intervention modulates microbiota-gut-nervous system [levels of Gamma-Aminobutiric acid (GABA) and short-chain fatty acids (acetate, propionate and butyrate)].
5. Evaluate changes associated with the dietary intervention in levels of neuropeptides (substance-P, neuropeptide-Y and Beta-Amyloid-42 and 40).
6. Evaluate if the dietary intervention produces changes in inflammatory markers (high-sensitivity C-reactive protein) and cytokine levels [interleukin-6 (IL-6) and tumor necrosis factor alpha (TNF-α)].
7. Determine changes associated with the dietary intervention in markers related to oxidative stress [advanced glycation end products (AGEs), carbonyl proteins and lipid peroxidation].

#### Blinding

Although allocation to the diet intervention was randomly assigned, the participant and the RD could not be blinded to diet. All other staff members involved in the measurement of any outcome were blind to the assignments, including the neuropsychologists, magnetic resonance imaging (MRI) technicians, and the lab technician where the blood tests were analyzed. Data entry was performed by a research assistant who was also blinded to group assignments.

#### Blood tests

Fasting blood samples were drawn and analyzed locally for fasting glucose, fasting insulin, glycated hemoglobin (HbA1c), high-sensitivity C-reactive protein (hsCRP), lipid profile, creatinine, homocysteine, folate, and cobalamin (B12). In addition, blood tubes were centrifuged, and plasma and serum were separated into tubes stored at −80°C for future analysis. Urine and feces samples were also obtained and stored at −80°C.

#### Anthropometric measurements and clinical scales

Anthropometric parameters were measured by trained dietitians using calibrated scales (BF511 body composition analyzer/scale, OMROM, Japan) and a wall-mounted stadiometer (Seca 242, HealthCheck Systems, Brooklyn, NY, USA). Waist circumference was measured midway between the lowest rib and the iliac crest. Body mass index (BMI) was calculated as weight per square meter (kg/m^2^). Fat-free mass, fat mass and visceral fat were measured by bioelectrical impedance analysis technique (BF511 body composition analyzer/scale, OMROM, Japan). Each measurement was made three times and the average value was calculated. Blood pressure (BP) was determined after a resting period of 10 min in the supine position using an automatic and calibrated sphygmomanometer (OMRON M3, OMRON Healthcare Europe, Spain). As indicated for the anthropometrical measures, BP was measured three times with a 1-min gap between each measurement and an average value was calculated. Smoking habits, Interview for Deterioration of Daily Living in Dementia (IDDD), Physical Functional Ability Questionnaire (FAQ5) and Neuropsychiatric Inventory–Questionnaire (NPI-Q) were assessed.

#### Clinical status

Regular medical visits to ascertain the existence of any changes in clinical characteristics or drug therapy were carried out (Table 2). Additional (“on demand”) visits were performed, when the patients attending the dietary visits reported any modification in their health status or treatment.

#### Comprehensive neuropsychological assessment

A neuropsychological assessment was performed by an experienced neuropsychologist specializing in the cognitive assessment of older adults. A paper-and-pencil battery, administered in face-to-face sessions, included commonly used cognitive tests presenting a range of cognitive domains, such as attention, executive functions, language, visuospatial, and memory (Supplementary Table 1).

##### Alzheimer’s disease assessment scale-cognitive

Changes in the Alzheimer’s disease Assessment Scale–Cognitive Subscale (ADAs-Cog-11) (11-task version) was the main outcome of our study (28) (Supplementary Table 1). ADAs-Cog-11 is a rating scale to assess the severity of cognitive dysfunction from mild to severe that includes 11 tasks, subject-completed tests and observer-based assessments. Memory, language, and praxis cognitive domains were assessed by this test (28).

##### Neuropsychological test battery

Alzheimer’s disease assessment scale-cognitive scores generally appear able to detect differences in cognitive ability in groups defined by an exposure that is expected to be associated with cognitive ability, although the magnitude of the differences detected tends to be small (28, 52). Responsiveness of the ADAs-Cog-11 to diverse treatment effects appears low compared with other global outcome measures designed to assess subdomains of cognition or other aspects of dementia and MCI syndromes (28). For this reason, other neuropsychological tests were also assessed to detect more subtle differences in MCI evolution in specific domains (i.e., memory, attention, executive, and visuospatial) and subdomains of cognition. A more detailed summary of the different neuropsychological tests performed is presented in Supplementary Table 2. With the aim to evaluate changes in both global neuropsychological condition and at each specific domain, different scores were calculated, using the information from these neuropsychological tests, grouping them by a total sum of values and dividing them by the number of tests used for each case (i.e., Σ Neuropsychological tests/number of Neuropsychological tests).

#### Functional neuroimaging studies by ^18^F-deoxy-2-[fluorine-18] fluoro-D-glucose-positron emission tomography

At baseline and at the end of each dietary intervention (i.e., at 24, 48, and 72-weeks), functional neuroimaging studies by 2-Deoxy-2-[fluorine-18] fluoro-D-glucose (FDG) positron emission tomography–computed tomography (PET/CT) were performed. The CT-based attenuation correction was carried out using the Siemens CAREDose 4D AEC system [Biograph mCT S (20) 3R system, Siemens Medical Solutions USA, Inc.].

## Statistical methods

All statistical analyses were performed with SPSS version 19.0 for Windows (SPSS Inc., Chicago, IL, USA). Data are presented as the mean ± standard error of the mean (SEM) for continuous variables and as proportions for the categorical variables.

### Analytic plan for results of diet effects on cognitive capacity

#### Primary statistical analysis

This study will be analyzed under the principle of intention-to-treat (ITT). The ITT analysis will include all randomized participants, regardless of any protocol deviation including non-adherence. Statistical comparisons will be performed using 2-sided significance tests. The primary statistical comparison will be analysis of variance (ANOVA) for repeated measures test with Bonferroni’s adjustment. To adjust for heterogeneity among the subjects, several baseline covariates, including age, sex, basal glucose levels, systolic and diastolic blood pressure, body mass index, diet consumed and drug therapy will be used. The level of significance for all the analyses will be a two-sided *p* < 0.05.

#### Secondary statistical analysis

Categorical variables will be analyzed by chi squared test, whereas continuous variables by ANOVA. Linear and logistic regression analyses will be performed to determine which variables studied were associated with changes in the progression of MCI through time. In order to detect more subtle differences in MCI evolution in specific domains and subdomains of cognition a neuropsychological test battery was assessed, and different scores were calculated grouping them by a total sum of values and dividing them by the number of tests used for each case (i.e., Σ Neuropsychological tests/number of Neuropsychological tests).

Analysis of variance for repeated measures test with Bonferroni’s adjustment and linear mixed effect models will be also used by biochemical characteristics, anthropometric measures, clinical scales and food intake (FFQ, 3 Days Register Food Consumption and MEDAS).

Based on the heterogeneity of the secondary outcomes, statistical tests will be selected individually for each outcome. For example, the processing and analysis of the series images will be performed using the free software SPM and the statistical package R (SPM12 (Statistical Parametric Mapping, Welcome Trust Center for Neuroimaging, London, UK)^1^ (53). PETs from each subject will be separately analyzed using SPM12. Single subject analysis and two multi subject analysis will be performed for testing for the main effects of subject and diet and testing for the main effects of subject and time, respectively. In addition, the gut microbiota changes, induced by the different dietary interventions, will be analyzed in term of the structure and composition. 16S metagenomic row data sequences will be analyzed using the Quantitative Insights into Microbial Ecology (QIIME2) program.^2^ These analyses, which include the relative abundance at different levels (phylum, class, order, etc.) and alpha and beta diversity metrics. Further, data modeling (Lasso, Random Forest, General lineal models, etc.) will allow us to evaluate any relationship between gut microbiota and cognitive changes and their influence in cognitive capacity. The level of significance for all the analyses will be a two-sided *p* < 0.05. Heterogeneity among the subjects will be adjusted for several baseline covariates, including age, and sex.

## Results

Participants’ baseline characteristics are presented in Tables 4, 5. The mean age was 73.1 ± 0.9, 57.4% of patients were males and 40.4% have ever smoked (10.6% being current smokers). Furthermore, mean HbA1c was 5.9 ± 0.1% (in a prediabetic range), whereas fasting glucose and insulin levels were 99.7 ± 3.3 mg/dL and 10.4 ± 0.9 mU/L (both within a normal range), respectively. With regards to the lipid profile, mean total cholesterol levels were 180 ± 5.1 mg/dL, triglycerides 99.8 ± 4.3 mg/dL, LDL-cholesterol 80.7 ± 5.0 mg/dL and HDL-cholesterol 57.1 ± 2.0 mg/dL. Mean systolic BP was 136 ± 2.1 mmHg and mean hsCRP levels were 8.8 ± 0.9 mg/dL (Table 4). Patients were overweight with a mean BMI of 27.9 ± 0.5 kg/m^2^ and mean waist circumference of 99.6 ± 1.6 cm.

**TABLE 4:** Baseline demographic, clinical and biochemical characteristics of the MCI patients.

Variable	MCI population (*n* = 47)	
	
	Mean	SEM
Age (years)	73.1	0.9
**Gender (%)**		
Male	57.4	
Female	42.6	
**Smoking (%)**		
Never	59.6	
In the past	29.8	
Current	10.6	
Diastolic blood pressure (mmHg)	73.6	1.5
Systolic blood pressure (mmHg)	136	2.1
**Blood tests**		
Fasting glucose (mg/dL)	99.7	3.3
Fasting insulin (mU/L)	10.4	0.9
HbA1c (%)	5.8	0.1
LDL-cholesterol (mg/dL)	80.7	5.0
HDL-cholesterol (mg/dL)	57.1	2.0
Total cholesterol (mg/dL)	180	5.1
Triglycerides (mg/dL)	99.8	4.3
Apolipoprotein A (mg/dL)	114.7	7.1
Apolipoprotein B (mg/dL)	143	3.8
Creatinine (mg/dL)	0.90	0.04
hsCRP (mg/dL)	8.8	0.9
Homocysteine (μmol/L)	22.1	1.8
Folic acid (μg/L)	11.4	0.8
Vitamin B12 (ng/mL)	403	27

Data are mean (standard error) or percentage of participants.

MCI, mild cognitive impairment; HbA1c, glycated hemoglobin; hsCRP, high sensitive C-reactive protein; SEM, standard error of the mean.

**TABLE 5:** Baseline anthropometric characteristics and treatment regimens of the MCI patients.

Variable	Total MCI population (*n* = 47)	
	
	Mean	SEM
Weight (kg)	72.8	0.9
BMI (kg/m^2^)	27.9	0.5
Fat-free mass (%)	28.2	0.6
Fat mass (%)	33.2	1.3
Visceral fat (%)	12.9	0.5
Waist circumference (cm)	99.6	1.6
Hip circumference (cm)	102	1.1

**Medication use**		

***Antihypertensive drugs (%)***		
ACE inhibitors	10.6	
ARB	36.2	
Calcium channel blockers	14.9	
***Lipid-lowering drugs (%)***		
Statins	55.3	
***Antidiabetics (%)***		
Metformin	17.0	
Insulin	10.6	
***MCI drugs (%)***		
Donepezil	25.5	
Rivastigmine	4.30	
Somazina	17.0	
Benzodiazepine	44.7	
Antidepressants	38.3	

Data are mean (standard error) or percentage of participants.

MCI, mild cognitive impairment; SEM, standard error of the mean; BMI, body mass index; ACE inhibitors, angiotensin-converting enzyme inhibitors; ARB, angiotensin-receptor blockers.

More than 50% of the patients were on antihypertensive and lipid-lowering (particularly, statins) drug therapy, whereas 27.6% of them were receiving antidiabetic treatment (Table 5).

Table 6 shows the baseline values of energy and nutrient intake. Mean energy intake was 2067 ± 74 kcal, while the percentage of total energy by carbohydrates, protein, and fat energy were 42.9 ± 1.1, 14.8 ± 0.4, 39.7 ± 1.1, respectively. Adherence to the Mediterranean diet as defined with the MEDAS was 7.51 ± 0.29 points (the score ranged from 0 to 14 points) at the baseline for the whole patient population (i.e., regardless of the randomization group to which they were assigned).

**TABLE 6:** Baseline values in energy and nutrient intake.

Variable	Total MCI population (*n* = 47)	
	
	Mean	SEM
Energy (Kcal)	2067	74
Total carbohydrates (% energy)	42.9	1.1
Fiber (g)	24.8	1.6
Total protein (% energy)	14.8	0.4
Total fat (% energy)	39.7	1.1
MUFAs (% energy)	48.1	2.7
PUFAs (% energy)	13.9	0.9
SFAs (% energy)	22.7	1.2
Adherence to the Mediterranean diet (MEDAS) (points)	7.51	0.29

MCI, mild cognitive impairment; SEM, standard error of the mean; MUFA, monounsaturated fatty acids; PUFA, polyunsaturated fatty acids; SFA, saturated fatty acids; MEDAS, 14-item Mediterranean diet adherence screener -range between 0 (minimum) and 14 (maximum) points.

## Discussion

The present manuscript describes the methodology, study participant selection, recruitment, adherence strategies and baseline characteristics of a randomized clinical study assessing the efficacy of a combined nutritional intervention (i.e., a Mediterranean diet rich in EVOO supplemented with probiotics (*Lactobacillus rhamnosus* and *Bifidobacterium longum*)], on cognitive capacity in patients with MCI.

Considering the fact that there are currently no effective pharmacological treatments for MCI (54), lifestyle modifications (such as physical activity and an improvement of diet quality) have shown promising results in slowing the progression of MCI (55). Previous studies focused on single nutrients and foods (ginko biloba), vitamins (vitamin E, C, and B12) or supplements (multivitamins) have reported inconsistent results (56–58). However, strong evidence exists for a beneficial effect of the Mediterranean diet [a dietary pattern rich in plant-based foods such as vegetables, whole grains, nuts, and olive oil [mainly VOO and EVOO) as the main source of fat (in particular MUFA)] in reducing the risk of developing cognitive impairment and dementia (19–22). Different pathways and underlying biological mechanisms have been proposed to explain the effects of the Mediterranean diet on cognitive impairment. In this context, adherence to a Mediterranean diet was associated with less AD biomarkers abnormalities (such as lower Pittsburgh compound B -PET deposition and higher brain glucose metabolism) in middle-aged adults (59, 60). Moreover, the Mediterranean diet reduces cardiovascular risk factors, which are themselves risk factors for the development of cognitive impairment (61, 62). This dietary pattern may also decrease oxidative stress and inflammation, thus potentially exerting neuroprotective properties (63–66). In particular, polyphenols and other minor components of VOO and EVOO showed a beneficial effect on β-aggregation, neurofibrillary tangles, autophagy and mitochondrial function, as well as in cerebral insulin resistance (67). Finally, it has been suggested that the Mediterranean diet could act as a modulator of the gut microbiota (68); the latter being also implicated in aging (69). Recent evidence supports a strong relationship between cognitive impairment disorders and gut microbiota alterations (67, 70). This association is based on the role of the gut-brain axis as a bidirectional communication pathway between the brain and the gastrointestinal tract (11, 12).

In this regard, oral probiotic consumption may modulate the capacity of the gut microbiota, by increasing the diversity and number of beneficial microbes, thus potentially leading to changes in the integrity of the intestinal barrier and the production of microbiota-derived metabolites, as well as to reduction of inflammation and oxidative stress (71) and alterations in the hypothalamic–pituitary–adrenal axis (72, 73). Therefore, some probiotics have been suggested as strategies for modulation of the central nervous system that could prevent cognitive decline, as well as attenuate or improve cognitive impairment related to dementia (74). However, there are certain limitations that do not permit the extraction of safe conclusions in relation to the effects of probiotics on cognitive function. First, clinical studies are mostly performed in patients with AD, with only a few of them conducted in patients with MCI. Secondly, although there are randomized controlled trials, a large number of them were double-blind (75). Moreover, some studies did not provide the exact probiotic strain(s) or the dose administered. Additionally, other studies lack of clarity regarding any form of power calculation to determine sample size or providing basic information such as age range and gender distribution (75, 76).

In this context, further well-designed, randomized controlled trials, with a primary focus on cognitive performance and potential mechanisms of action, are required to elucidate how effective probiotic interventions can be for improving cognitive function. To the best of our knowledge, the present clinical study (a randomized, double-blind, and controlled dietary intervention trial with probiotic supplementation), would be the first to evaluate the synergistic action of two different dietary strategies with potential effects on cognitive capacity in patients with MCI, i.e., a Mediterranean diet (well-known for its cardioprotective, anti-inflammatory and antioxidant properties) with probiotic supplementation (with *Lactobacillus rhamnosus* and *Bifidobacterium longum*).

The main limitations of the present study are those inherent to all long-term intervention studies. In this context, although several layers of internal controls have been established to ascertain adherence to the diet, the nature of the study contributes to potential deviations from a strict dietary adherence. On the other hand, the cost of acquiring certain foods (such as fish or nuts) that are determinant for following a Mediterranean diet could be a limitation for an adequate adherence to this type of diet. In conclusions, we described the methods, study participant selection and recruitment, adherence strategies and baseline characteristics of a randomized, latin-square crossover, double-blind, and controlled dietary intervention trial, performed in MCI patients, assessing the efficacy of a combined nutritional intervention on cognitive capacity through the modulation of pathways and mechanisms related to the *gut-brain axis*. This clinical study will also emphasize the need to evaluate MCI participants and provide dietary therapeutic strategies, for clinical and individual practice, focusing on reducing their risk to develop AD.

## Data availability statement

Data is available upon request to the corresponding author.

## Ethics statement

The studies involving human participants were reviewed and approved by the Ethics Committee of Reina Sofía University Hospital (trial protocol 1496/27/03/2009). The patients/participants provided their written informed consent to participate in this study.

## Author contributions

PP-M and FP-J contributed to the study concept. PP-M, JL-M, NK, JD-L, and EY-S critically reviewed the manuscript. MC and EY-S contributed to the design of the manuscript, figure preparation, edition, and manuscript drafting. MC, ACo, AL-A, GQ-N, JA-D, OR-Z, ACa, CC-G, CC-M, JV-C, EC-A, JO-S, and EA-M contributed to the acquisition and analysis of data. All authors gave final approval for all aspects of the work, agreed to be fully accountable for ensuring the integrity and accuracy of the work, and read and approved the final manuscript.

## Footnotes

1
http://www.fil.ion.ucl.ac.uk/spm

2
https://qiime2.org/

## References

[1] Langa K Levine D. The diagnosis and management of mild cognitive impairment: a clinical review. *JAMA.* (2014) 312:2551–61. 10.1001/jama.2014.13806 25514304 PMC4269302

[2] Sanford A. Mild cognitive impairment. *Clin Geriatr Med.* (2017) 33:325–37. 10.1016/j.cger.2017.02.005 28689566

[3] Lee H Kim D Lee W Kim H Kim Y. Preventive approach for overcoming dementia. *Arch Pharm Res.* (2019) 42:647–57. 10.1007/s12272-019-01168-3 31187441

[4] Alzheimer’s Association. 2015 Alzheimer’s disease facts and figures. *Alzheimers Dement.* (2015) 11:332–84. 10.1016/j.jalz.2015.02.003 25984581

[5] Pandya S Clem M Silva L Woon F. Does mild cognitive impairment always lead to dementia? A review. *J Neurol Sci.* (2016) 369:57–62. 10.1016/j.jns.2016.07.055 27653867

[6] Hampel H Hardy J Blennow K Chen C Perry G Kim S The Amyloid-beta pathway in Alzheimer’s disease. *Mol Psychiatry.* (2021) 26:5481–503. 10.1038/s41380-021-01249-0 34456336 PMC8758495

[7] Villemagne V Burnham S Bourgeat P Brown B Ellis K Salvado O Amyloid beta deposition, neurodegeneration, and cognitive decline in sporadic Alzheimer’s disease: a prospective cohort study. *Lancet Neurol.* (2013) 12:357–67. 10.1016/S1474-4422(13)70044-9 23477989

[8] Venigalla M Sonego S Gyengesi E Sharman M Munch G. Novel promising therapeutics against chronic neuroinflammation and neurodegeneration in Alzheimer’s disease. *Neurochem Int.* (2016) 95:63–74. 10.1016/j.neuint.2015.10.011 26529297

[9] Tiwari S Atluri V Kaushik A Yndart A Nair M. Alzheimer’s disease: pathogenesis, diagnostics, and therapeutics. *Int J Nanomedicine.* (2019) 14:5541–54. 10.2147/IJN.S200490 31410002 PMC6650620

[10] Lowe V Wiste H Senjem M Weigand S Therneau T Boeve B Widespread brain tau and its association with ageing, Braak stage and Alzheimer’s dementia. *Brain.* (2018) 141:271–87. 10.1093/brain/awx320 29228201 PMC5837250

[11] Clark A Mach N. The crosstalk between the gut microbiota and mitochondria during exercise. *Front Physiol.* (2017) 8:319. 10.3389/fphys.2017.00319 28579962 PMC5437217

[12] Cryan J O’Riordan K Cowan C Sandhu K Bastiaanssen T Boehme M The microbiota-gut-brain axis. *Physiol Rev.* (2019) 99:1877–2013. 31460832 10.1152/physrev.00018.2018

[13] ZhaoXYuanLFengLXiYYuHMaW Association of dietary intake and lifestyle pattern with mild cognitive impairment in the elderly. *J Nutr Health Aging.* (2015) 19:164–8. 10.1007/s12603-014-0524-2 25651441

[14] An R Liu G Khan N Yan H Wang Y. Dietary habits and cognitive impairment risk among oldest-old chinese. *J Gerontol B Psychol Sci Soc Sci.* (2019) 74:474–83. 10.1093/geronb/gbw170 28184889

[15] Wang Z Pang Y Liu J Wang J Xie Z Huang T. Association of healthy lifestyle with cognitive function among Chinese older adults. *Eur J Clin Nutr.* (2021) 75:325–34. 10.1038/s41430-020-00785-2 33116235

[16] Ajith TA. A recent update on the effects of omega-3 fatty acids in Alzheimer’s disease. *Curr Clin Pharmacol.* (2018) 13:252–60. 10.2174/1574884713666180807145648 30084334

[17] Williams D Hagg S Pedersen N. Circulating antioxidants and Alzheimer disease prevention: a mendelian randomization study. *Am J Clin Nutr.* (2019) 109:90–8. 10.1093/ajcn/nqy225 30596810 PMC6358036

[18] Dhakal S Kushairi N Phan C Adhikari B Sabaratnam V Macreadie I. Dietary polyphenols: a multifactorial strategy to target Alzheimer’s disease. *Int J Mol Sci.* (2019) 20:5090. 10.3390/ijms20205090 31615073 PMC6834216

[19] Singh B Parsaik A Mielke M Erwin P Knopman D Petersen R Association of mediterranean diet with mild cognitive impairment and Alzheimer’s disease: a systematic review and meta-analysis. *J Alzheimers Dis.* (2014) 39:271–82. 10.3233/JAD-130830 24164735 PMC3946820

[20] Santoro A Pini E Scurti M Palmas G Berendsen A Brzozowska A Combating inflammaging through a Mediterranean whole diet approach: the NU-AGE project’s conceptual framework and design. *Mech Ageing Dev.* (2014) 136-137:3–13. 10.1016/j.freeradbiomed.2013.08.109 24342354

[21] Coelho-Junior H Trichopoulou A Panza F. Cross-sectional and longitudinal associations between adherence to Mediterranean diet with physical performance and cognitive function in older adults: a systematic review and meta-analysis. *Ageing Res Rev.* (2021) 70:101395. 10.1016/j.arr.2021.101395 34153553

[22] Garcia-Casares N Gallego Fuentes P Barbancho M Lopez-Gigosos R Garcia-Rodriguez A Gutierrez-Bedmar M. Alzheimer’s disease, mild cognitive impairment and mediterranean diet. A systematic review and dose-response meta-analysis. *J Clin Med.* (2021) 10:4642. 10.3390/jcm10204642 34682764 PMC8537524

[23] Perez-Martinez P Huelgas R Perez-Jimenez F. Healthy planetary diet: do we have to rethink the recommendations based on the Mediterranean diet? *Clin Investig Arterioscler.* (2019) 31:218–21. 10.1016/j.artere.2019.10.002 31585614

[24] Romero-Cabrera J Yubero-Serrano E Diaz-Caceres A Serran-Jimenez A Arenas-Montes J Alcala-Diaz J Educational strategy to improve cardiovascular health and mitigate food insecurity: rationale for the E-DUCASS program. *Span J Med.* (2022) 2:1–8. 10.24875/SJMED.21000025

[25] Abraham D Feher J Scuderi G Szabo D Dobolyi A Cservenak M Exercise and probiotics attenuate the development of Alzheimer’s disease in transgenic mice: role of microbiome. *Exp Gerontol.* (2019) 115:122–31. 10.1016/j.exger.2018.12.005 30529024

[26] Rezaeiasl Z Sepehri G Salami M. Probiotic treatment improves the impaired spatial cognitive performance and restores synaptic plasticity in an animal model of Alzheimer’s disease. *Behav Brain Res.* (2019) 376:112183. 10.1016/j.bbr.2019.112183 31472194

[27] Rezaeiasl Z Salami M Sepehri G. The effects of probiotic lactobacillus and bifidobacterium strains on memory and learning behavior, long-term potentiation (LTP), and some biochemical parameters in beta-amyloid-induced rat’s model of Alzheimer’s disease. *Prev Nutr Food Sci.* (2019) 24:265–73. 10.3746/pnf.2019.24.3.265 31608251 PMC6779093

[28] Kueper J Speechley M Montero-Odasso M. The Alzheimer’s disease assessment scale-cognitive subscale (ADAS-COG): modifications and responsiveness in pre-dementia populations. A narrative review. *J Alzheimers Dis.* (2018) 63:423–44. 10.3233/JAD-170991 29660938 PMC5929311

[29] Skinner J Carvalho J Potter G Thames A Zelinski E Crane P The Alzheimer’s disease assessment scale-cognitive-plus (ADAS-Cog-Plus): an expansion of the ADAS-Cog to improve responsiveness in MCI. *Brain Imaging Behav.* (2012) 6:489–501. 10.1007/s11682-012-9166-3 22614326 PMC3873823

[30] Morris J. The clinical dementia rating (CDR): current version and scoring rules. *Neurology.* (1993) 43:2412–4. 10.1212/WNL.43.11.2412-a 8232972

[31] Morris J. Clinical dementia rating: a reliable and valid diagnostic and staging measure for dementia of the Alzheimer type. *Int Psychogeriatr.* (1997) 9(Suppl. 1):173–6. 10.1017/S1041610297004870 9447441

[32] Berg L. Clinical dementia rating (CDR). *Psychopharmacol Bull.* (1988) 24:637–9. 3249765

[33] Blesa R Pujol M Aguilar M Santacruz P Bertran-Serra I Hernandez G Clinical validity of the ‘mini-mental state’ for Spanish speaking communities. *Neuropsychologia.* (2001) 39:1150–7. 10.1016/S0028-3932(01)00055-0 11527552

[34] Hobson V Hall J Humphreys-Clark J Schrimsher G O’Bryant S. Identifying functional impairment with scores from the repeatable battery for the assessment of neuropsychological status (RBANS). *Int J Geriatr Psychiatry.* (2010) 25:525–30. 10.1002/gps.2382 19862695

[35] Randolph C Tierney M Mohr E Chase T. The repeatable battery for the assessment of neuropsychological status (RBANS): preliminary clinical validity. *J Clin Exp Neuropsychol.* (1998) 20:310–9. 10.1076/jcen.20.3.310.823 9845158

[36] Koenig H Meador K Cohen H Blazer D. Self-rated depression scales and screening for major depression in the older hospitalized patient with medical illness. *J Am Geriatr Soc.* (1988) 36:699–706. 10.1111/j.1532-5415.1988.tb07171.x 3042842

[37] Navarro-Lopez V Martinez-Andres A Ramirez-Bosca A Ruzafa-Costas B Nunez-Delegido E Carrion-Gutierrez M Efficacy and safety of oral administration of a mixture of probiotic strains in patients with psoriasis: a randomized controlled clinical trial. *Acta Derm Venereol.* (2019) 99:1078–84. 31453631 10.2340/00015555-3305

[38] Sanchez Macarro M Avila-Gandia V Perez-Pinero S Canovas F Garcia-Munoz A Abellan-Ruiz M Antioxidant effect of a probiotic product on a model of oxidative stress induced by high-intensity and duration physical exercise. *Antioxidants.* (2021) 10:323. 10.3390/antiox10020323 33671691 PMC7926771

[39] Caviglia G Tucci A Pellicano R Fagoonee S Rosso C Abate M Clinical response and changes of cytokines and zonulin levels in patients with diarrhoea-predominant irritable bowel syndrome treated with bifidobacterium longum es1 for 8 or 12 weeks: a preliminary report. *J Clin Med.* (2020) 9:2353. 10.3390/jcm9082353 32717980 PMC7464152

[40] Quintana-Navarro G Alcala-Diaz J Lopez-Moreno J Perez-Corral I Leon-Acuna A Torres-Pena J Long-term dietary adherence and changes in dietary intake in coronary patients after intervention with a Mediterranean diet or a low-fat diet: the CORDIOPREV randomized trial. *Eur J Nutr.* (2020) 59:2099–110. 10.1007/s00394-019-02059-5 31342228

[41] Astrup A Bertram H Bonjour J de Groot L de Oliveira Otto M Feeney E WHO draft guidelines on dietary saturated and trans fatty acids: time for a new approach? *BMJ.* (2019) 366:l4137. 10.1136/bmj.l4137 31270106

[42] World Health Organization [WHO]. *Diet, nutrition and the prevention of chronic diseases. World health organization technical report series.* (Vol. 916). Geneva: World Health Organization (WHO) (2003). p. 1–149. 12768890

[43] World Health Organization [WHO]. *WHO guidelines approved by the guidelines review committee. Guideline: sugars intake for adults and children.* Geneva: World Health Organization (2015).

[44] World Health Organization [WHO]. *W guidelines approved by the guidelines review committee. Guideline: sodium intake for adults and children.* Geneva: World Health Organization (2012).

[45] Fernandez-Ballart J Pinol J Zazpe I Corella D Carrasco P Toledo E Relative validity of a semi-quantitative food-frequency questionnaire in an elderly Mediterranean population of Spain. *Br J Nutr.* (2010) 103:1808–16. 10.1017/S0007114509993837 20102675

[46] Martin-Moreno J Boyle P Gorgojo L Maisonneuve P Fernandez-Rodriguez J Salvini S Development and validation of a food frequency questionnaire in Spain. *Int J Epidemiol.* (1993) 22:512–9. 10.1093/ije/22.3.512 8359969

[47] Moreiras OCA Cabrera L Cuadrado C. *Tablas de composición de alimentos y guía de prácticas.* Madrid: Ediciones Pirámide (2013).

[48] Mataix JGL Mañas M Martinez de Victoria E Llopis J. *Tabla de composición de alimentos españoles.* Granada: Universidad de Granada (2003).

[49] Taggart N. Diet, activity and body-weight. A study of variations in a woman. *Br J Nutr.* (1962) 16:223–35. 10.1079/BJN19620024 13918930

[50] Castro D. [Psychological aspects of treatment compliance in the insulin- dependent diabetic child]. *Ann Pediatr.* (1991) 38:455–8. 1952702

[51] Schroder H Fito M Estruch R Martinez-Gonzalez M Corella D Salas-Salvado J A short screener is valid for assessing Mediterranean diet adherence among older Spanish men and women. *J Nutr.* (2011) 141:1140–5. 10.3945/jn.110.135566 21508208

[52] Karin A Hannesdottir K Jaeger J Annas P Segerdahl M Karlsson P Psychometric evaluation of ADAS-COG and NTB for measuring drug response. *Acta Neurol Scand.* (2014) 129:114–22. 10.1111/ane.12153 23763450

[53] Friston KJ Holmes A Worsley K Poline J Frith C Frackowiak RS. Statistical parametric maps in functional imaging: a general linear approach. *Human Brain Mapping.* (1994) 2:189–210. 10.1002/hbm.460020402

[54] Cooper C Ketley D Livingston G. Systematic review and meta-analysis to estimate potential recruitment to dementia intervention studies. *Int J Geriatr Psychiatry.* (2014) 29:515–25. 10.1002/gps.4034 24706605

[55] Sofi F Valecchi D Bacci D Abbate R Gensini G Casini A Physical activity and risk of cognitive decline: a meta-analysis of prospective studies. *J Intern Med.* (2011) 269:107–17. 10.1111/j.1365-2796.2010.02281.x 20831630

[56] KrauseDRoupasP. Effect of vitamin intake on cognitive decline in older adults: evaluation of the evidence. *J Nutr Health Aging.* (2015) 19:745–53. 10.1007/s12603-015-0539-3 26193858

[57] Snitz B O’Meara E Carlson M Arnold A Ives D Rapp S Ginkgo biloba for preventing cognitive decline in older adults: a randomized trial. *JAMA.* (2009) 302:2663–70. 10.1001/jama.2009.1913 20040554 PMC2832285

[58] Naeini A Elmadfa I Djazayery A Barekatain M Ghazvini M Djalali M The effect of antioxidant vitamins E and C on cognitive performance of the elderly with mild cognitive impairment in Isfahan, Iran: a double-blind, randomized, placebo-controlled trial. *Eur J Nutr.* (2014) 53:1255–62. 10.1007/s00394-013-0628-1 24326981

[59] Berti V Walters M Sterling J Quinn C Logue M Andrews R Mediterranean diet and 3-year Alzheimer brain biomarker changes in middle-aged adults. *Neurology.* (2018) 90:e1789–98. 10.1212/WNL.0000000000005527 29653991 PMC5957301

[60] Walters M Sterling J Quinn C Ganzer C Osorio R Andrews R Associations of lifestyle and vascular risk factors with Alzheimer’s brain biomarker changes during middle age: a 3-year longitudinal study in the broader New York City area. *BMJ Open.* (2018) 8:e023664. 10.1136/bmjopen-2018-023664 30478117 PMC6254410

[61] Yubero-Serrano E Lopez-Moreno J Gomez-Delgado F Lopez-Miranda J. Extra virgin olive oil: more than a healthy fat. *Eur J Clin Nutr.* (2019) 72(Suppl. 1):8–17. 10.1038/s41430-018-0304-x 30487558

[62] Torres-Pena J Rangel-Zuniga O Alcala-Diaz J Lopez-Miranda J Delgado-Lista J. Mediterranean diet and endothelial function: a review of its effects at different vascular bed levels. *Nutrients.* (2020) 12:2212. 10.3390/nu12082212 32722321 PMC7469011

[63] Iadecola C Duering M Hachinski V Joutel A Pendlebury S Schneider J Vascular cognitive impairment and dementia: JACC scientific expert panel. *J Am Coll Cardiol.* (2019) 73:3326–44. 10.1016/j.jacc.2019.04.034 31248555 PMC6719789

[64] Yubero-Serrano E Garcia-Rios A Delgado-Lista J Delgado-Casado N Perez-Martinez P Rodriguez-Cantalejo F Postprandial effects of the Mediterranean diet on oxidant and antioxidant status in elderly men and women. *J Am Geriatr Soc.* (2011) 59:938–40. 10.1111/j.1532-5415.2011.03381.x 21568967

[65] Lopez-Moreno J Quintana-Navarro G Delgado-Lista J Garcia-Rios A Delgado-Casado N Camargo A Mediterranean diet reduces serum advanced glycation end products and increases antioxidant defenses in elderly adults: a randomized controlled trial. *J Am Geriatr Soc.* (2016) 64:901–4. 10.1111/jgs.14062 27100598

[66] Delgado-Lista J Alcala-Diaz J Torres-Pena J Quintana-Navarro G Fuentes F Garcia-Rios A Long-term secondary prevention of cardiovascular disease with a Mediterranean diet and a low-fat diet (CORDIOPREV): a randomised controlled trial. *Lancet.* (2022) 399:1876–85. 35525255 10.1016/S0140-6736(22)00122-2

[67] Silva P Rodriguez-Perez M Gomez-Torres O Burgos-Ramos E. Olive oil and wine as source of multi-target agents in the prevention of Alzheimer disease. *Nutr Res Rev.* (2021). [Epub head of print]. 10.1017/S095442242100041X 34895363

[68] Rejeski J Wilson F Nagpal R Yadav H Weinberg R. The impact of a mediterranean diet on the gut microbiome in healthy human subjects: a pilot study. *Digestion.* (2021) 103:133–40. 10.1159/000519445 34749376 PMC8916822

[69] Ctoi A Corina A Katsiki N Vodnar D Andreicut A Stoian A Gut microbiota and aging-A focus on centenarians. *Biochim Biophys Acta Mol Basis Dis.* (2020) 1866:165765. 10.1016/j.bbadis.2020.165765 32169505

[70] Jiang C Li G Huang P Liu Z Zhao B. The gut microbiota and Alzheimer’s disease. *J Alzheimers Dis.* (2017) 58:1–15. 10.3233/JAD-161141 28372330

[71] Den H Dong X Chen M Zou Z. Efficacy of probiotics on cognition, and biomarkers of inflammation and oxidative stress in adults with Alzheimer’s disease or mild cognitive impairment - a meta-analysis of randomized controlled trials. *Aging.* (2020) 12:4010–39. 10.18632/aging.102810 32062613 PMC7066922

[72] Smith C Emge J Berzins K Lung L Khamishon R Shah P Probiotics normalize the gut-brain-microbiota axis in immunodeficient mice. *Am J Physiol Gastrointest Liver Physiol.* (2014) 307:G793–802. 10.1152/ajpgi.00238.2014 25190473 PMC4200314

[73] Farzi A Frohlich E Holzer P. Gut microbiota and the neuroendocrine system. *Neurotherapeutics.* (2018) 15:5–22. 10.1007/s13311-017-0600-5 29380303 PMC5794709

[74] Xiao J Katsumata N Bernier F Ohno K Yamauchi Y Odamaki T Probiotic bifidobacterium breve in improving cognitive functions of older adults with suspected mild cognitive impairment: a randomized, double-blind, placebo-controlled trial. *J Alzheimers Dis.* (2020) 77:139–47. 10.3233/JAD-200488 32623402 PMC7592675

[75] Ruiz-Gonzalez C Roman P Rueda-Ruzafa L Rodriguez-Arrastia M Cardona D. Effects of probiotics supplementation on dementia and cognitive impairment: a systematic review and meta-analysis of preclinical and clinical studies. *Prog Neuropsychopharmacol Biol Psychiatry.* (2021) 108:110189. 10.1016/j.pnpbp.2020.110189 33285265

[76] Eastwood J Walton G Van Hemert S Williams C Lamport D. The effect of probiotics on cognitive function across the human lifespan: a systematic review. *Neurosci Biobehav Rev.* (2021) 128:311–27. 10.1016/j.neubiorev.2021.06.032 34171323

