# Supplementary material for: Effect of the Mediterranean diet and probiotic supplementation in the management of mild cognitive impairment: Rationale, methods, and baseline characteristics
Source: Front Nutr. 2022 Dec 8;9:1037842. doi: 10.3389/fnut.2022.1037842 (PMC9773830; doi:10.3389/fnut.2022.1037842)
Supplement: Supplementary file 1 [file Data_Sheet_1.docx]

**Supplementary Table 1.** Cognitive assessments: ADAS-Cog-11 and its domains.

| **ADAS-COG-11** | **Domain** | **Subdomain** |
| --- | --- | --- |
| Word Recall | Memory | Language |
| Commands | Executive | Language |
| Constructional Praxis | Executive | Executive |
| Naming Objects and Fingers | Memory | Language |
| Ideational Praxis | Executive | Executive |
| Orientation | Memory | Orientation |
| Word Recognition | Memory | Language |
| Remembering Test Instructions | Memory | Language |
| Comprehension of Spoken Language | Attention | Language |
| Word Finding Difficulty | Memory | Language |
| Language | Memory | Language |

**Supplementary Table 2.** Other neuropsychological tests battery assessments and their domains

| **Domain** | **Subdomain** | **Test** |
| --- | --- | --- |
| ***Memory*** | ***Memory*** | Free and Cued Selective Reminding test (FCSRT)   - Inmediate memory (free + facilitated) - Delayed memory (free + facilitated) |
|  | ***Memory and working memory*** | Rey-Osterrieth Complex Figure Test (RCFT)   - Immediate memory   Delayed memory |
|  | ***Working memory*** | CORSI block-tapping test   - Direct - Inverse - Letter number sequencing (LNS) |
|  | ***Language and working memory*** | TOKEN test |
| ***Attention*** | ***Processing speed*** | Stroop test   - Reading - Denomination - Interference   Trail Making Test   - Part A   Symbol Digit Modalities test |
|  | ***Alternate attention*** | Trail Making Test   - Part B |
| ***Executive*** | ***Executive function*** | Rey-Osterrieth Complex Figure Test (RCFT)   - Copy   Tower of London-DX (TOL-DX) test   - Correct - Movements - Execution - Resolution   Verbal fluidity   - Words - Animals |
|  | ***Inhibition*** | Tower of London-DX (TOL-DX) test   - Latency |
| ***Visuospatial*** | ***Spatial Cognition*** | Visual object and space perception (VOSP) battery   - Number location - Position discrimination   Benton Visual Retention |
|  | ***Perception*** | Visual object and space perception (VOSP) battery   - Object decision - Progressive silhouette |
